# Effect of Traditional Chinese Medicine on the Cardiovascular Diseases

**DOI:** 10.3389/fphar.2022.806300

**Published:** 2022-03-21

**Authors:** Yang Jiang, Qi Zhao, Lin Li, Shumin Huang, Shuai Yi, Zhixi Hu

**Affiliations:** ^1^ College of Traditional Chinese Medicine, Hunan University of Chinese Medicine, Changsha, China; ^2^ Hunan Academy of Traditional Chinese Medicine Affiliated Hospital, Changsha, China

**Keywords:** traditional chinese medicine, cardio vascular disease, oxidative stress, antioxidant, natural product

## Abstract

**Background:** Traditional Chinese medicine (TCM) is the health care system developed with the help of clinical trials that are based ideally on the scientific model of regulation.

**Objective:** This systematic health care system relies on some specific unique theories and practical experiences to treat and cure diseases, thus enhancing the public’s health.

**Review Methodology:** The current review covers the available literature from 2000 to 2021. The data was collected from journals research articles, published books, thesis, and electronic databases, search engines such as Google Scholar, Elsevier, EBSCO, PMC, PubMed, ScienceDirect, Willey Online Library, Springer Link, and CNKI) searching key terms, cardiovascular disease, traditional Chinese medicines, natural products, and bioactive compounds. Full-length articles and abstracts were screened for the collection of information included in the paper.

**Results:** Clinical trials on the TCM and basic research carried out on its mechanism and nature have led to the application and development of the perfect design of the research techniques, for example, twofold striking in acupuncture that aid in overcoming the limitations and resistances in integrating and applicability of these experiences and trials into the pre-existing biomedical models. Furthermore, TCM has also been utilized from ancient times to treat heart diseases in Asia, particularly in China, and is now used by people in many other areas. Cardiovascular disease (CVD) is mainly developed by oxidative stress. Hence antioxidants can be beneficial in treating this particular disease. TCM has a wide variety of antioxidant components.

**Conclusion:** The current review article summarizes the underlying therapeutic property of TCM and its mechanism. It also overviews the evidence of the mechanism of TCM action in CVD prevention by controlling oxidative stress and its signaling pathway.

## Introduction

According to the WHO, the leading cause of death is coronary heart disease (CHD), with 7.4 million deaths in 2013 and one-third of all deaths (Kelly et al., 2012; van Halewijn et al., 2017; Yang et al., 2017). By 2021, CHD, the most severe and prevalent menace to human life, is expected to continue (Kaur et al., 2008). The multifactorial nature of heart disease involves complex interactions between physiological, genetic, and environmental factors (Ahmad and Bhopal, 2005). It is multifactorial and entails complex interactions between physiology, genetics. The environment Diabetes, hypertension, smoking, and hyperlipidemia have all been found to have been linked to oxidative stress in previous studies, including traditional risk factors for CHD. Although oxidative stress was related to the development of coronary artery disease (CAD) (Juni et al., 2013), some studies have investigated the markers of oxidative stress (OS) and found that they can predict the evolution of CHD. OS is one of the risk factors for CAD and has been shown to affect the prediction and survival of CAD patients (Juni et al., 2013; Li et al., 2018). Various animal atherosclerosis and coronary artery disease models have shown that the development of oxidative stress is closely related. If the ability of the antioxidant system to minimize reactive oxygen and other free radicals is insufficient, it may experience oxidative stress. Reactive oxygen (ROS) species have been shown to cause damage to DNA and proteins in deoxyribonucleic acid (DNA) and protein levels, which harm the structure and function of the cardiovascular system (Lubos et al., 2011) if oxidative stress occurs. Patients with CAD had elevated levels of ROS in their imagery discovered by imagery. The mediated apoptosis of mitochondria in cells and smooth muscles is induced by the production of excessive ROS in endothelial and smooth cells, resulting in imbalances between cells’ antioxidant ability and the surrounding prooxidant ability to promote and increase the development of coronary heart conditions (Tullio et al., 2013; Zhang et al., 2015). The three most important antioxidant systems in the body are SOD, catalase (CAT), Glutathione peroxidase (GSH-PX), and they all work together to protect the body from damage.

In addition, numerous molecular antioxidants, including -carotene, ascorbic acid, -tocopherol, and reduced glutathione (GSH), are essential for human health (Leopold, 2015) and are critical for human health (GSH). The risk of CHD has been reduced effectively by antioxidant therapy (Willcox et al., 2008). Inpatients’ oxidative status records are often insufficient, and specific antioxidants are seldom prescribed, affecting the treatment’s effectiveness. On the other side, China has a long and distinguished history, dating back thousands of years, of TCM, which is increasingly popular worldwide, particularly for the treatment of CVD (Wang et al., 2018a). Many Chinese herbal extracts with antioxidant effects have been discovered (LI et al., 2011). These extracts have proved effective in preventing the cardiovascular disease from developing. This review aims to summarize the sources, effect, mechanism of TCM action, and current practices in the inhibition of CVD by modulating different pathways.

## Understanding (Overview) Therapeutic Effect of Chinese Medicine (TCM)

TCM origins are still debated, although evidence suggests it has existed for about 5000 years. Old acupuncture needles and herbal therapy rests demonstrate it lasts between 4,000 and 8,000 years (Ma, 2000; Pan et al., 2014). The Yijing (also known as the I Ching or the Book of Changes) and Huangdi Neijing (also known as the Yellow Emperor’s Classic of Internal Medicine) were the earliest known TCM philosophical and clinical texts. It depicts the flow of life using a mathematical model of regulation as a base. The Huangdi Neijing is as important as the Hippocratic Corpus (Sertel et al., 2010). It describes its modifications and modes and provides emotional guidance.

**FIGURE 1:**
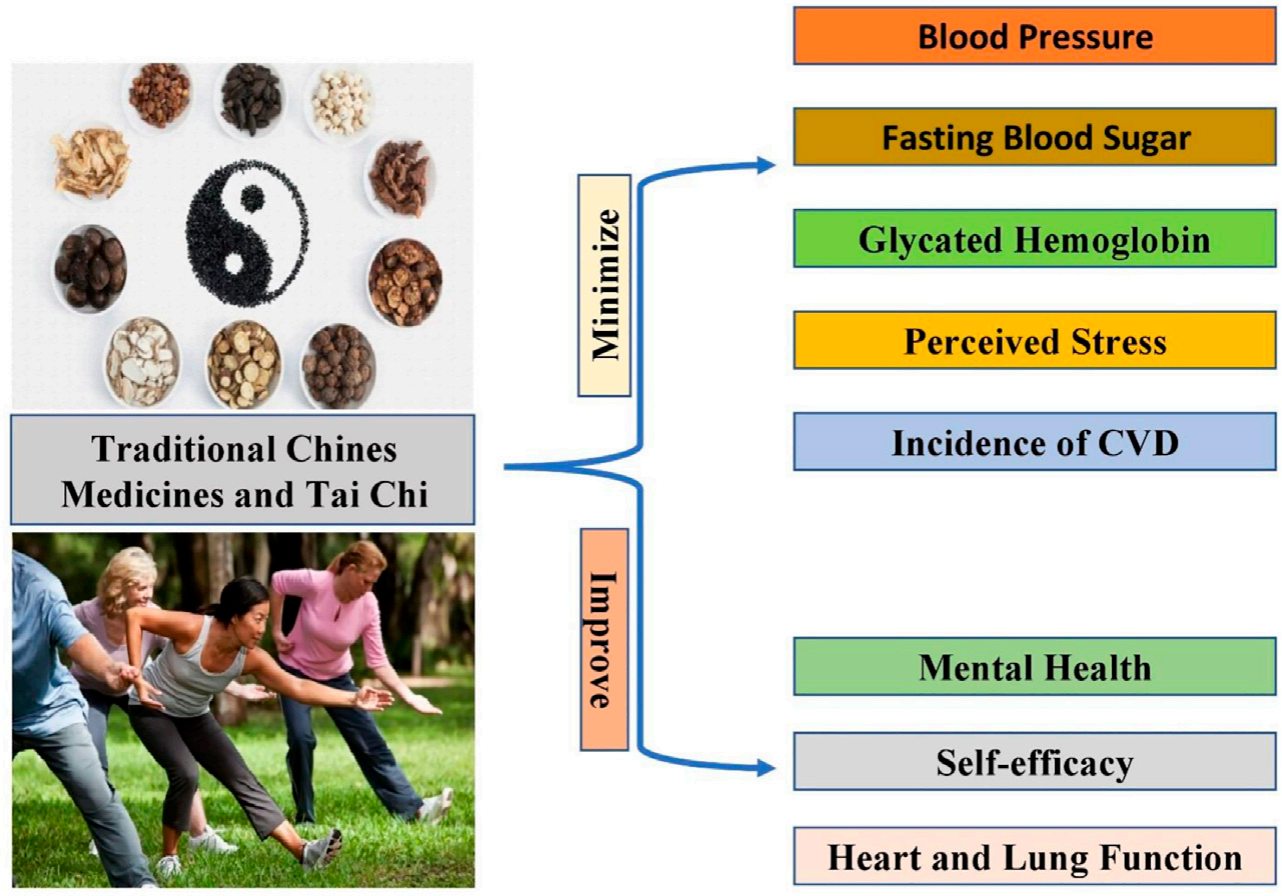
Shows the therapeutic potential of TCM and tai chi. Mechanism of improving health conditions and minimizing the risk of disease.

The five phases of transgression (elements) and Yin and Yang are basic TCM ideas (“Elements”). Their main activities are acupuncture, moxibustion, Chinese herbs and diets, and Tai Chung, Tuina, Qigong, and Taijiquan. It describes the effects and functional powers of body functions like “qi,” “blood,” and “Xue,” as well as the consequences and differences in the diagnosis of “jin ye” syndromes. The ancient Chinese doctors believed everything was the same substance,’ or ‘qi.’ Because everything is symbiotically connected via the “qi” system, this ideology emphasizes oneness and wholeness. Traditional Chinese medicine aims to balance the effects of the body’s qi, also called the Vital Force and live-in harmony with the environment’s qi. There are energies emitted by the Sun and the moon and the energy emitted by plants, soil, water, animals, and natural forms (Johnson et al., 2000). TCM treats both secondary (“Biao”) and primary (“ben”) aspects of chronic and acute ailments. TCM is used in gynecology, traumatology, internal medicine, external medicine, pediatrics, dermatology, otolaryngology, emergency medicine (Greten, 2013). Table 1 Enlists some of the medicine that is used in treating several kinds of diseases. The therapeutic potential of TCM and tai chi in improving health conditions and minimizing the risk of CVD shown in Figure 1.

**TABLE 1:** different Chinese medicine used for the treatment of various condition.

Ingredients	Indication	Source of ingredients	References
Semen cuscutae chinensis	History of miscarriages in early	*Cuscuta chinensis Lam*	Yang et al. (2016)
Radix codonopsis	Scanty menstruation, uterine bleeding, miscarriage, infertility	*Codonopsis pilosula*	Li et al. (2014)
Dehydrotumulosic acid	Modulating the immune system of the body, invigorating the spleen and tranquilizing the mind, excreting dampness, inducing diuresis	*Poria cocos* (Schw.)	Song et al. (2002)
β-glucans, ridoids, proleic substances,l-arabinose, d-xylose, D-glucuronic	Nutrition, Cardiovascular Diseases, Diabetics	Hordeum vulgare L	Emilia-Ancuța et al. (2019)
MTB (MTB70)	Cardiovascular, cerebrovascular effects, the antioxidative, neuroprotective, antifibrotic, anti-inflammatory, and antineoplastic activities, anti-cancer activity	*S. miltiorrhiza*	(Leung et al., 2010; Wang and Yeung, 2011; Zaid et al., 2017)

The concept of “yin and yang” is crucial in Chinese medicine. It denotes linguistic and cultural connections in Chinese. It has several symbolic meanings, as seen in the example above. The masculine, heaven, fire, strong, light, day, summer, and the outward, active are examples of “YIN.” Yin is a Chinese character. The Five Phases (Wu Xing) are Wood, Fire, Earth, Metal, and Water. For each bigram, the sign symbols are two trigrams representing the “orbs” or signs. This part of the cycle is identified by visible markers and symptoms of an individual’s functional condition. This concept can be used in many fields, including anatomy, physiology, and psychology (Liu, 2011): The Five Phases have long been known to interact physically (the sheng and ke cycles) and pathologically (the cheng, wu, and sheng cycles). Disrupting the five-phase balance increases the risk of pathogenic symptoms (Focks et al., 2008). The Compass helps us navigate life. The dynamics of the rose is a circular function of vegetative regulation with components of “yin” and “yang” and abscissa and ordinate axes of Earth and Sun phases. Metal signifies a relative energy scarcity and cyclical energy distribution; water represents renewal, and earth drives metamorphosis. Both the parasympathetic (vagal) and sympathetic (nervous) processes are more active during the yang phases (Greten, 2007). The sympathetic, parasympathetic, and enteric nervous systems are all linked to the vegetative system. For example, when the enteric nervous system is stressed, it is less active. During downregulation and fire and metal, the enteric nervous system is more involved in regaining energy. Aside from that, RAAS may integrate muscular tonus and motion patterns employing hypertonic, hyper-dynamic, hypotonic, and hypo-dynamic functional practices in the “yin” phase domains (Matos et al., 2021). In Traditional Chinese Medicine, physiological activity, pathological changes, and an organ’s relationship to essential constituents (Qi, Xue, Jin Yi, etc.) are more critical than morphological structure (TCM). As per Western medical belief, anatomical structure is crucial in ‘judging exterior appearance from inside.’ According to TCM, after assessing “inner from the outer,” one studies the body’s outside appearance and changes, assuming “inner appears on outer.” In Western medicine, symptom perception evolves into illness perception, leading to a deeper grasp of disease nature. Rather than diseases, the expression and separation of syndromes are critical in traditional Chinese medicine. Yin Yang syndromes are signs of pathogenic causes that disrupt visceral function and yin yang equilibrium. Tongue and pulse diagnostics are examples of indications that can be discovered in various methods. Multiple Western diseases may be the same as some TCM “syndromes,” and some TCM “syndromes” incorporate Western diseases (Cheung, 2000). In Chinese medicine, the essence of the kidney, or “jing,” is a fundamental principle of ageing. Each person is born with a certain amount of “jing,” which they gradually use throughout their lives. Some specialists compare “kidney deficiency syndrome” to “ageing” in Western Medicine. Heart qi failure was also linked to heart failure (Dong, 2013). The Chinese term “zang-fu” refers to both morphological and functional entities of the human body. It separates the internal organs into two groups based on yin-yang phases: solid organs (heart and pericardium, liver, spleen, lung, kidney) and hollow organs (small intestine and triple burner, gallbladder, stomach, large intestine, and bladder) (Liu, 2011). TCM meridians, or channels, and collateral in connective tissue are divided into jing Luo networks. These channels are links between acupoints that alter the clinical signs of various orbs. Each of the hand’s three “yang” meridians and the foot’s three “yang” meridians are Yin. They are related to the “zang” organs and the “fu” organs. This causes “qi” and “Xue” to circulate cyclically throughout the body (Liu, 2011).

## Yin and Yang, Tai Chi and CVD

**FIGURE 2:**
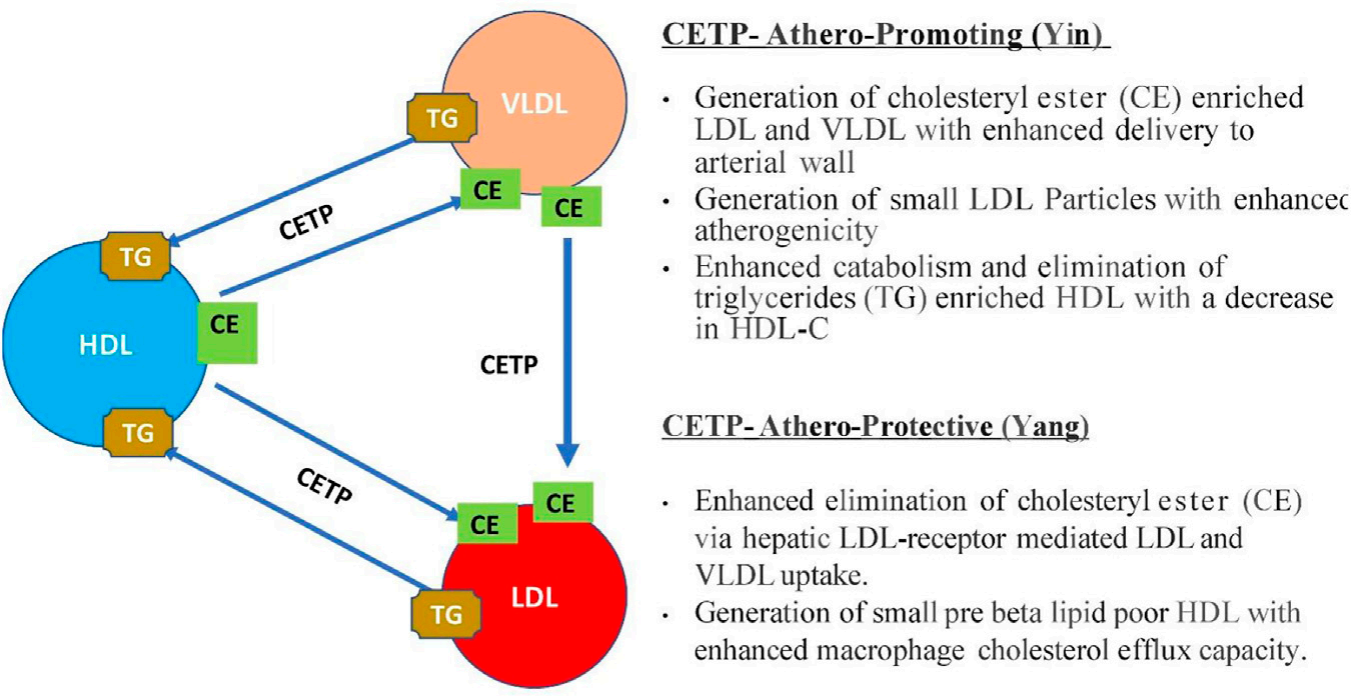
Shows the Yin and Yang of CETP in atherosclerosis. CE transfer from HDL particles to LDL-VLDL particles is facilitated by CETP in exchange for TG and CETP also facilitates CE movement from VLDL to LDL particles.

Cholesteryl ester transfer protein (CETP) facilitates the transfer of cholesteryl ester from high-density lipoprotein (HDL) particles to low-density lipoprotein (LDL)-very-low-density lipoprotein (vLDL) particles in exchange for triglycerides, thereby participating in reverse cholesterol transport and regulating circulating HDL cholesterol (HDL-C) levels (Shah, 2009). HDL-C has evolved as an attractive target for the prevention of CVD (Leança et al., 2010). Adipokines are involved in a yin-yang homoeostatic balance whereby there are substantial benefits (Mattu and Randeva, 2013). Adiponectin, omentin, and apelin have cardioprotective effects. Resistin, leptin, adipocyte fatty acid-binding protein, and visfatin are pro-inflammatory with a negative impact on cardiovascular function (Smekal and Vaclavik, 2017). CETP activity is associated with increased atherosclerosis progression and/or cardiovascular risk (Shah, 2009). The Yin and Yang of CETP in atherosclerosis are shown in Figure 2.

A study reported that the TCM constitutions including Yang-deficiency, Qi-deficiency, and Yin-deficiency were significantly associated with coronary artery disease (CAD). As compared with Neutral participants, participants with the TCM constitutions had a higher prevalence of CAD (Xu et al., 2018). Stress and a sedentary lifestyle are major determinants of CVD. As tai chi involves exercise and can help in stress reduction, it may be effective in the primary prevention of CVD (Hartley et al., 2014). A study report that Tai Chi is a suitable exercise for obese people, regulates blood pressure, improves cardiac and pulmonary function, minimizes the prevalence of CVD and other chronic diseases, helping better life standard and quality. As compared to the control group (38.33%) the prevalence of cardiovascular and cerebrovascular disease in the Tai Chi group was lower (16.67%) (Sun et al., 2019). Tai Chi minimizes many CVD risk factors and improves psychosocial well-being. Tai Chi, significantly reduce BP (systolic −12.46 mmHg; diastolic −3.20 mmHg), fasting blood sugar (−1.27 mmol/L), glycated hemoglobin (−0.56%), lower perceived stress (−2.32 score), and improved perceived mental health (+3.54 score) and exercise self-efficacy (+12.83 score). Tai Chi can be suggested as an important exercise for spending a CVD-free healthy life (Chan et al., 2018).

## Use of Traditional Chinese Medicine in Cardiovascular Disease

### Myocardial Infarction Role of ROS and Adipokines

MI is one of the most frequent forms of ischemic heart disease and accounts for a large percentage of mortality. Increasing evidence demonstrates that ROS cause cell death following MI and are linked to MI initiation. Reducing ROS levels may be an important therapeutic goal in MI-induced injury. It has been found that producing ROS with various antioxidants can help prevent oxidative stress-related harm and improve MI status (Bagheri et al., 2016). Adipose tissues are important energy storage organs and also act as active endocrine glands making many important chemical messengers and bioactive proteins known as adipokines. Adipose tissue working status is signaled to the brain and other target organs by protein peptides known as adipokines (Shibata et al., 2017). Several adipokines have been discovered in the past 20 years. In impairment of adipose tissue, secretion of most adipokines is altered and upregulated leading to obesity. Obesity leads to the development of CVD. Adipokines such as interleukin (IL-6), tumor necrosis factor (TNF-α), IL-1β, and resistin are pro-inflammatory and intensify various metabolic and CVD (Lau et al., 2017). Adipokines are involved in a ‘good-bad’, yin-yang homoeostatic balance whereby there are substantial benefits (Mattu and Randeva, 2013). Some adipokines such as adiponectin, omentin, and apelin have anti-inflammatory, angiogenesis, reducing hypertension, atherosclerosis, and cardioprotective effects. Others (visfatin, leptin, resistin, adipocyte fatty-acid-binding protein) are pro-inflammatory with a negative impact on cardiovascular function (Smekal and Vaclavik, 2017). Table 2 summarizes the role of the TCM in regulating ROS.

**TABLE 2:** Reducing ROS in myocardial infraction by TCM.

TCM type	Molecular formula	Study condition	Plant source	Mode of action	Sources
Bioactive Component of TCM	C_21_H_20_ O_11_ Astragalin; Ophiopogonin D (C_44_H_70_O_16_)	*Vivo*	*Rosa agrestis Cuscuta chinensis*	Anti-inflammatory, antipoetic, and antioxidative	Qu et al. (2016)
	Salvianolic acid (C_36_H_30_O_16_; C_26_H_22_O_10_), tanshinone (C_18_H_12_O_3_; C_19_H_18_O_3_)	*Vivo*	*Radix S. miltiorrhiza* (*Danshen* **)**	Regulating factors that participate in oxidative stress and apoptosis further inhibits the release of intracellular calcium and prevents the adhesion pathway of the cell	Wang and Yeung, (2011)
	Curcumin (C_21_H_20_O_6_)		*Curcuma Longa*	Activation of the signaling pathway of JAK2/STAT3 that suppress and prevent oxidative damage and myocardium apoptosis	Liu et al. (2017)
	Oxysophoridine (C_15_H_24_N_2_O_2_		*Sophora flavescens*	Anti-inflammatory, antipoetic, and antioxidative	Meng et al. (2015)
	Danshensu (C_9_H_10_O_5_)		*Pueraria lobata Ohwi*	Akt/ERK1/2/Nrf2 signaling pathway activation	Yu et al. (2015)
	Azafrin (C_27_H_38_O_4_)	*Vivo* and *Vitro*	*Alectra parasitica*	Nrf2-ARE signaling pathway activation	Yang et al. (2017)
	Punicalagin (C_48_H_28_O_30_)	*Vivo*	*Punica granatum*	AMPK activation	Ding et al. (2017)
	Xanthones (C_13_H_8_O_2_)	*vivo*	-	antipoetic, and antioxidative	Liu et al. (2017)
	Barbaloin (C_21_H_22_O_9_)		*Aloe Emodin Anthrone*	Antioxidant and anti-inflammatory	Zhang et al. (2017)
Traditional Chinese medicine in patent drugs	Shenxian-shengmai	*Vivo*	*Catharanthus roseus*	SOD activity enhancement and GSH content aggrandization	Zhao et al. (2018a)
	Cardiotonic pill Danqi pill (DQP)	*Vivo*	*Salvia miltiorrhiza (SM), Panax notoginseng (PN),* and *Borneol*	NOX activities inhibition decreased TG, LDL, ApoB, increased and activate ApoA-I, cardiac FATP, CPTI levels	(Yang et al., 2013; Mao et al., 2015; Ma et al., 2019)
	Hongjingtian injection	*Vitro* and *Vivo*	*Rhodiola wallichiana*	Reducing oxidative damage to myocardial	(Chan et al., 2018) (Zhang et al., 2017)
	Guanxintai	*Vitro* and *Vivo*	*Calotropis procera*	Reduce or decrease the activity of NOX and MAPK	Yang et al. (2017)
	Dunye Guanxinning	*Vivo*	*Dioscorea zingiberensis*	Reduce or inhibit the activity of inflammasome with the help of AMPK Pathway	Zhang et al. (2018)

## Essential Bioactive Compounds and Ingredients of Traditional Chinese Medicine and CVD

1. *S. miltiorrhiza*: A well-known Chinese herbal medicine, *S. miltiorrhiza*, has been shown to help treat cardiovascular diseases (Si-yan et al., 2007). Several studies have shown enhanced small artery circulation (Zhao et al., 2010), reduced ROS production (Zhou et al., 2012; Guan et al., 2013), and cardiac protection against ischemia-reperfusion injury (Yin et al., 2013). *S. miltiorrhiza* extracts hydrophilic and lipophilic substances (SAL, C_36_H_30_O_16_; C_26_H_22_O_10_) and tanshinone (TAN, C_18_H_12_O_3_; C_19_H_18_O_3_). TAN (C_18_H_12_O_3_), using MI models (Wang and Yeung, 2011), investigated the cardio-protective effects of SAL and TAN in rats. Gene activity was discovered by microarray and verified by a reverse transcription-polymerase chain reaction in quantitative real-time (RT-PCR). Apoptotic and oxidative stress proteins are likely to be downregulated in SAL, while intracellular calcium and cell adhesion pathways in MI are likely to be upregulated. *S. miltiorrhiza* contributed to of inhibition soluble epoxide hydrolase (sEH), a pro-inflammatory enzyme based on an LC-MS/MS assay, and discovered that tanshinone IIA and cryptotanshinone were potent (the inhibition constant [K_i_] = 0.87 µM) and medium (K_i_ = 6.7 µM) mixed-type inhibitors of sEH, respectively, and Salvianolic acid C was a moderate (K_i_ = 8.6 µM) noncompetitive inhibitor of sEH (Huang et al., 2016).
2. Danshensu (C_9_H_10_O_5_), a critical water-soluble component of *S. miltiorrhiza*, was investigated. One study examined DSS’s cardio-protective abilities in an ischemia-reperfusion model (Hartley et al., 2014). The results showed that DSS increased endogenous antioxidants such GSH-PX, SOD, malondialdehyde, CAT, and heme oxygenase-1 (HO-1) activity and lowered creatine kinase and lactate blood dehydrogenase levels. Activation of the signaling pathways Akt/ERK1/2/Nrf2 has improved antioxidant defense (Zhang et al., 2017) (Tang et al., 2011). Through the Akt serine/threonine kinase (Akt)-endothelial nitric oxide synthase (eNOS) signaling pathway, *S. miltiorrhiza* and ligustrazine injection (SLI) show a good impact on myocardial ischemia/reperfusion (I/R) and hypoxia/reoxygenation (H/R) damage in mice exposed to coronary artery occlusion (Huang et al., 2016).
3. Flavonoid astragalin (C_21_H_20_O_11_) is found in *Rosa agrestis*, persimmon, and green tea. It comes from *R. agrestis* leaves, persimmons, or seeds of green tea (Cho et al., 2014). Many reports have shown that astragalin has anti-inflammatory, antioxidant, and other beneficial qualities. Qu *et al.* (Qu et al., 2016) used the Langendorff apparatus to assess astragalin’s cardioprotective properties against I/R heart injury. Pretreatment with astragalin improved myocardial function in the study subjects. The levels of MDA, TNF-alpha, intracellular ROS, and IL-6 significantly decrease in astragalin-treated groups. Astragalin’s anti-apoptotic, antioxidant, and anti-inflammatory properties make it cardioprotective (Zhou et al., 2015).
4. Ophiopogonin D (OP-D) is a monomeric component utilized for clinically relevant Shenmai injection (SEM-I). This molecule has been shown to have anti-apoptotic, antioxidant, and anti-inflammatory properties (Huang et al., 2015; You et al., 2016). The left anterior descending coronary artery was also occluded in rats to create the MI/R model for studying the antioxidants OP-ED and SM-I (Huang et al., 2018). An infarct is reduced in size, and heart architecture is improved by using OP-ED and SEM-I to protect cardiac function from MI/R. In both OP-ED and SM-I studies, the PI3K/eNOS signaling pathway and the NF-B signaling pathway were cardioprotective.
5. Curcumin (C_21_H_20_O_6_), produced from *Curcuma longa* L roots, is used as a flavoring and a traditional medicinal. It is used in both cooking and medicine. Many animals have exhibited anti-inflammatory, antioxidant, and anti-carcinogenic properties (Kumphune et al., 2015). According to one study, curcumin may have a protective effect on heart function in MI/R rats (Liu et al., 2017). The rats’ left anterior coronary artery descending was surgically closed. The existence of lipid peroxides and antioxidant enzymes in cardiac tissue was then studied. Curcumin appears to minimize the risk of CHD by activating JAK2/STAT3, decreasing oxidative damage, and decreasing myocardial apoptosis (Cho et al., 2013; Guo et al., 2014).
6. Pomegranate (*Punica granatum L*) juice contains the bioactive punicalagin molecule (C_48_H_28_O_30_), which exhibits antioxidant I/R injury protection (Yaidikar et al., 2014).
7. Antioxidative mechanisms have shown penicalagin (C_48_H_28_O_30_) neuroprotective benefits on I/R wounds. Another study (Ding et al., 2017) looked into PUN’s potential heart-protective mechanisms against MI/R damage. The MI/R used the anterior descending coronary artery connection (LADCA). PUN protects the heart from MI/R damage by improving cardiac function, decreasing serum creatine kinase-MB (CK-MB) and LDH, and preventing myocardial apoptosis. PUN protects against I/R-induced ROS and cardiac injury via stimulating AMPK monophosphate activation (Cao et al., 2015; Ding et al., 2017).
8. Barbaloin (BAR): Barbaloin (C_21_H_22_O_9_) is the main medicinal component found in *Aloe barbadensis miller*. It is a delicious herb with antioxidant properties. BAR’s cardio-protective effects in myocardial infarction were investigated by Zhang *et al.* Rats were intragastrically administered BAR before MI surgery. BAR administration decreased I/R-induced ROS and inflammation in the heart of rats and rats via activating AMPK activation (Zhang et al., 2017; Ren et al., 2018). Cui et al., experiments show that Bar pretreatment inhibits the expression of endoplasmic reticulum stress-associated proteins and CNPY2-positive cell apoptosis ratio of cardiomyocytes confirmed by immunohistochemistry. Bar pre-treatment may weaken MIRI by preventing the CNPY2-PERK apoptotic pathway (Cui et al., 2019).
9. Oxysophoridine (OSR) (C_15_H_24_N_2_O_2_), an alkaloid contained in the Chinese herbal remedy *Sophora alopecuroides* (L.), has been shown to reduce inflammation and apoptosis (Yu et al., 2013). A rat study explored OSR’s cardio-protective properties against myocardial infarction (MI) (Meng et al., 2015). OSR reduced myocardial enzymes such as CK-MB, cardiac troponin T, and lactate dehydrogenase in individuals. The antioxidant enzyme catalase and SOD and non-enzymatic oxidant glutathione increased in OSR-treated rats, while MDA decreased. OSR also inhibits the activity of inflammatory cytokines (Speir, 2000). OSR reduces myocardial damage in an AMI rat model, and its cardioprotective actions are mediated by anti-apoptotic, anti-inflammatory, and antioxidative mechanisms. Notable suppression of the inducible (iNOS), intercellular adhesion molecule-1, nuclear factor kappa B, and cyclooxygenase-2 was observed in OSR-treated mice. Marked reduction in the TNF-α, inflammatory-related protein prostaglandin E2, interleukin-1β (IL-1β), IL-6, and interleukin-8 (IL-8) was observed in the OSR-treated group (250 mg/kg) (Wang et al., 2015).
10. Xanthones (C_13_H_8_O_2_) is a plant compound that is extracted from the *Gentianella acuta,* used in Mongolian medicine to treat coronary artery disease (CAD). In Chinese medicine, it is called “Wenxincao” (or “Wenxincao” in simplified Chinese). Kuang *et al.* (Kuang et al., 2016), reported the effect of *G. acuta* on myocardial I/R damage in isolated rats. Several hemodynamic variables were measured during perfusion. These data reveal that *G. acuta* xanthones improve cardiac function, boost antioxidant enzymes like SOD (Superoxide dismutase), SDH (succinate dehydrogenase), CAT (catalase), MDH (Malate dehydrogenase), and the GSH/GSSG (Glutathione/glutathione disulfide) ratio, while decreasing activity of CK (Creatine kinase), MDA (Malondialdehyde) and LDH (Lactate dehydrogenase) levels. Also, xanthones can simultaneously upregulate Bcl-2 and downregulate Bax. In animal studies, *G. acuta* xanthones showed cardioprotective benefits on myocardial I/R injury via antioxidant and anti-apoptotic activities (Mahendran et al., 2014; Tantapakul et al., 2016).
11. Azafrin isolated from *Centeranthera Grandiflora Benth* is an old Chinese folk remedy for circulatory problems. One of YCDG’s most common active compounds is azafrin (C_27_H_38_O_4_) (Yang et al., 2018). As part of their research into azafrin’s potential myocardial preservation mechanisms, Yang *et al.* (Yang et al., 2018)*in vivo*, azafrin treatment improves cardiac function and reduces myocardial enzyme levels, cardiac troponin I (cTnI) and MDA, while increasing the antioxidant enzyme SOD activity. Overall, azafrin protects against myocardial injury by activating Nrf2-antitoxin.

## TCM Extract and CVD

Bao-Xin-Tang (BXT): A TCM remedy called BXT is used to treat CHD. You’ll find *Codonopsis pilosula, Atractylodes macrocephalus, Astragalus propinquus*, and *Fructus crataegi*. It has been demonstrated to improve blood flow and protect myocardium in MI patients (Kumphune et al., 2015) (Zhang et al., 2006). A study conducted by Wang *et al.* (Wang et al., 2018b), examined BXT’s cardioprotective effects against myocardial infarction. The rat model of MI was created by lining the rat’s anterior left coronary artery. Several studies have demonstrated that BXT can reduce infarction size, myeloperoxidase, IL-6, and CRP while increasing SOD and anti-inflammatory mediators such as interleukin 10 (IL-10). BXT has antioxidant and anti-inflammatory effects (Shang et al., 2013a). Dan-Shen Yin (DSY): DSY is a Chinese medicine kind. DSY is a famous Chinese herbal compound that contains herbs like *Fructus amomi* and *S. miltiorrhiza* (Liu, 2010). A study looked into DSY’s MI protection. The left anterior descending coronary artery branch was studied in rats for size, inflammatory factor concentrations, and antioxidant enzyme activity. A reduction in infarction, IL-6, CRP, TNF, and MAD was observed with DSY (Yan et al., 2012; Wang et al., 2018b). These findings show that DSY possesses anti-inflammatory and antioxidant properties that protect against ischemia in rats. The chemical structure of the compounds used as TCM is shown in Table 3.

**TABLE 3:** Showing chemical structure of the compounds used as TCM.

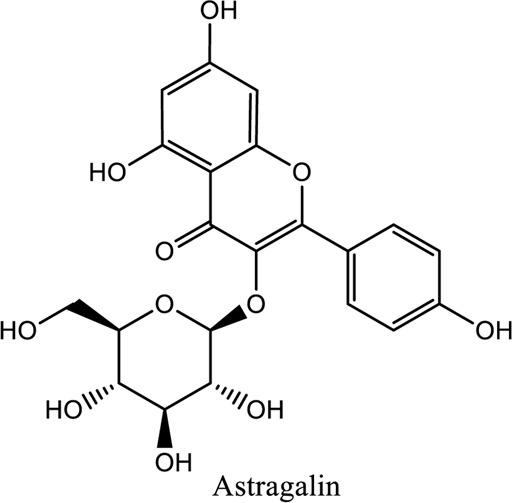	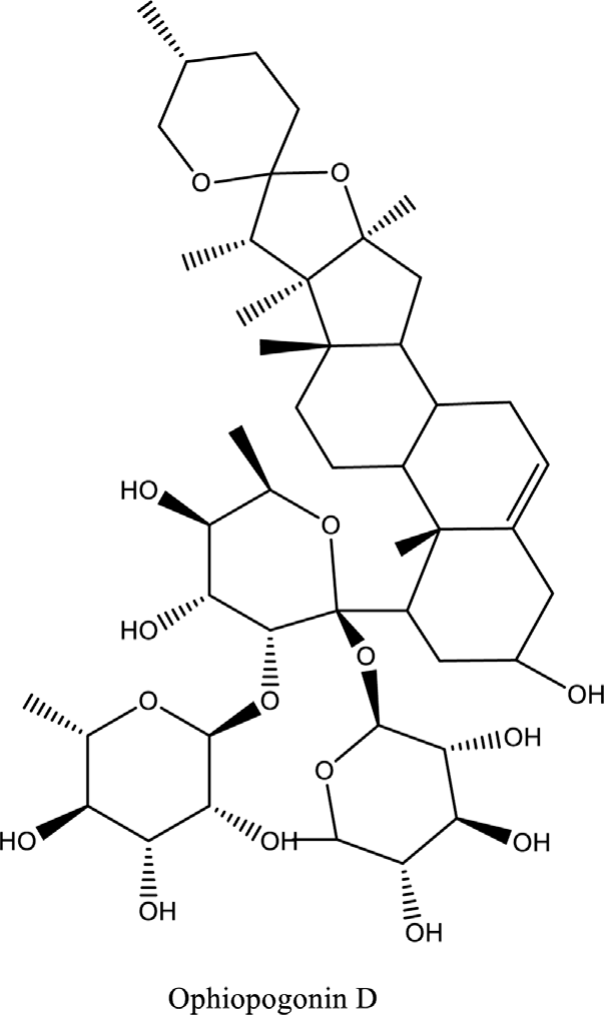
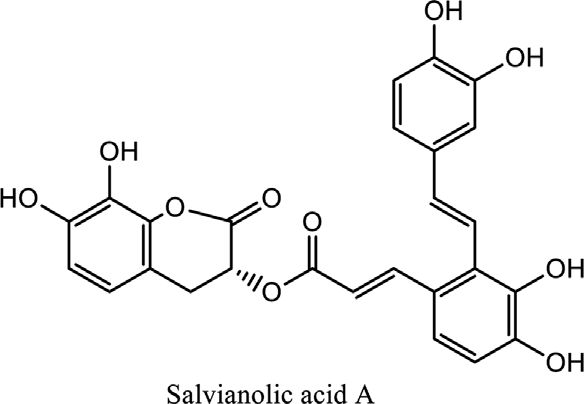	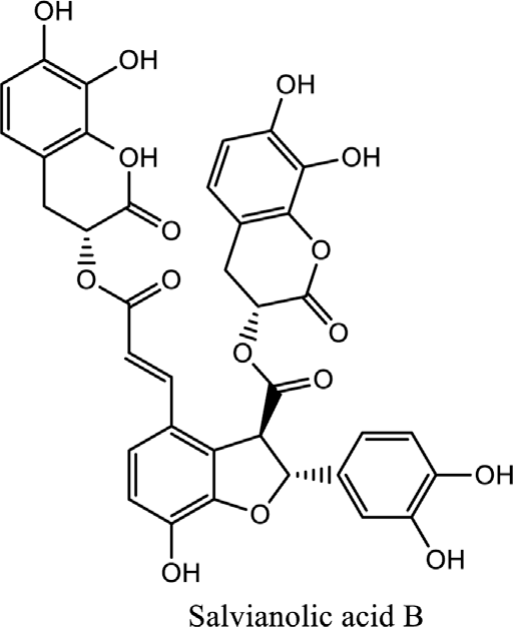
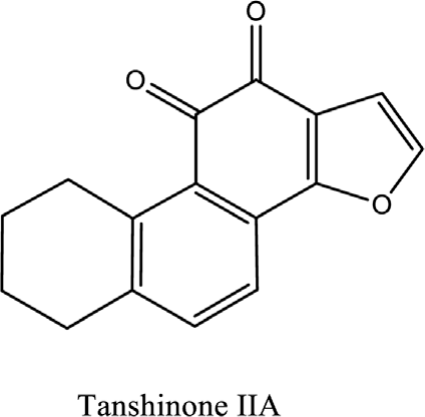	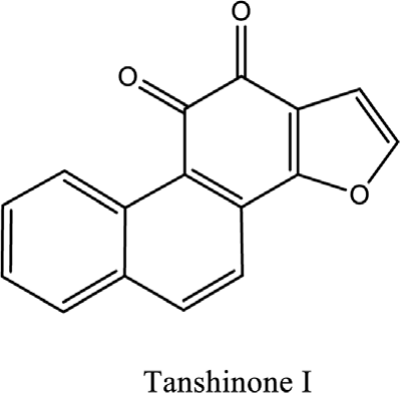
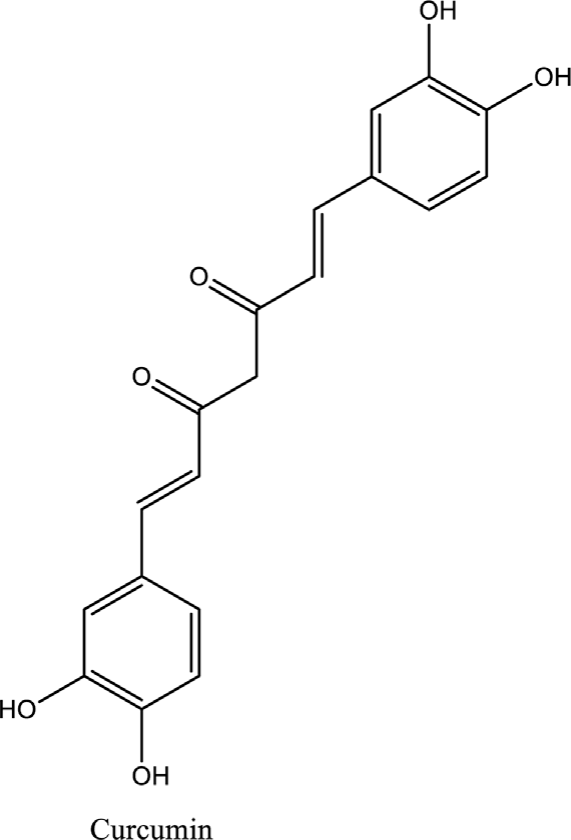	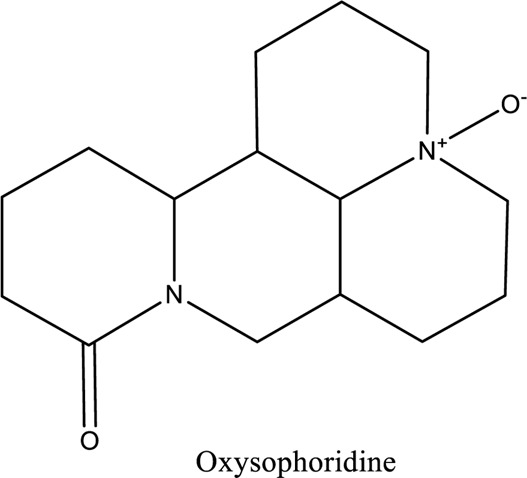
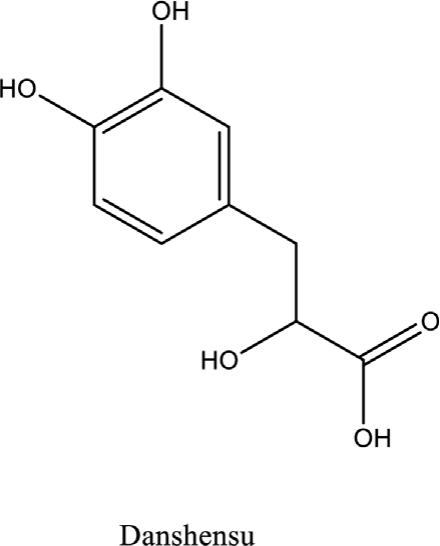	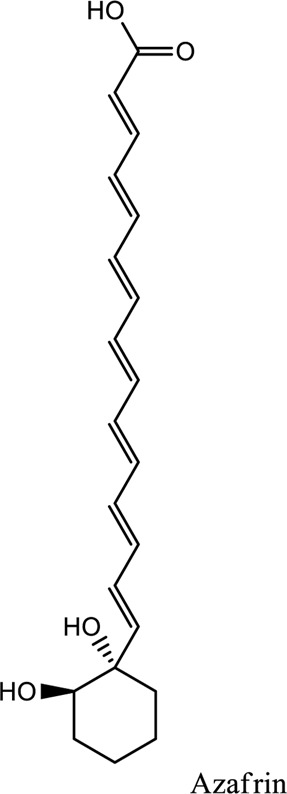
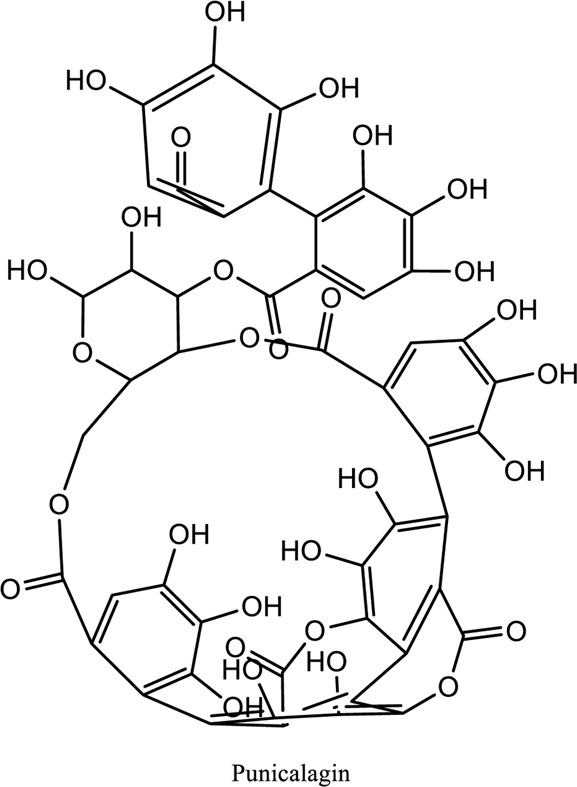	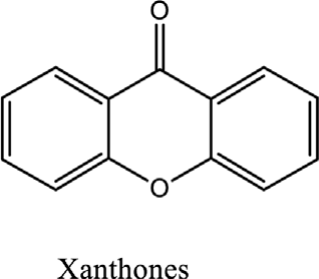
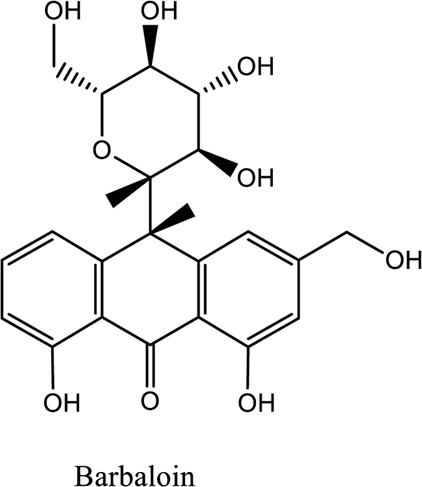	

## Use of Chinese Medicine in Patented Drugs

*Dunye Guanxinning* (DG): DG is used for angina, hyperlipidemia, and CHD treatment (Zhen et al., 2016; Yuan et al., 2018). DG is used to treat hypertension and Diabetes. In one study (Zhang et al., 2018), reducing caspase one activation and neutrophil infiltration reduced myocardial I/R damage. DG reduced neutrophil infiltration while decreasing beta (IL-1) interleukin. In rat cardiac tissue, Caspase-1 activity and AMPK activation inhibited DG. Thus, DG may block the AMPK pathway (Zhen et al., 2016; Zhang et al., 2018). It is derived from the herb *Rhodiola Rosea* and helps prevent vascular issues such as CVD and angina (Qi et al., 2016). Apoptosis-related proteins caspase three and Bcl-2 were changed in cells treated with HJT. HJT also altered the action of the Akt, ERK/mTOR, and Akt/Beclin-1 pathways, which were previously unknown. The HJT coronary ligation model showed reduced infarct size, improved heart function, and elevated protein light chain expression 3B (LC-3B). HJT improved myocardial damage by altering the apoptosis balance and autopathy and minimizing ROS (Shi et al., 2015). It is widely used in cardiology. Guanxintai (GXT), a TCM formula, is found in Ginseng (*Panax quinquefolius, L*), *Astragalus propinquus*, and *Ophiopogon* root. GXT has been demonstrated to have cardioprotective effects on angina (Song et al., 2012), arrhythmia (Qi et al., 2012), and blood lipids. Yang *et al.* (Yang et al., 2017), investigated GXT’s anti-ischemic and antioxidant properties on ischemic cardiomyocytes. The study found that GXT decreased myocardial and apoptotic cell injury and enhanced cardiac function after MI. GXT also reduced ROS production and NOX and MAPK enzyme expression in cells. The complement’s cardioprotective actions are responsible for the antioxidant NOX suppression in GXT (Guan et al., 2010). Heart pill: Heart med: The cardio tonic pill (CP) is extensively used to treat ischemic angina pectoris (abdominal pain). A study (Yang et al., 2013) indicated that the mechanisms behind CP’s antioxidant activity were favorable. Male rats were ligated and repurposed before slaughter. CP decreased myocardial damage, ROS, and microcirculation disturbances. CP reduced the protein expression of NOX subunits p67phox, gp91phox, and p47phox when given I/R. The CP has been shown to minimize NOX activity in I/R-induced rat heart damage and microcirculation disorders (Zhao et al., 2010; Yang et al., 2013). In clinical practice, SXSM oral fluid, a Chinese formula chemical, has been used for bradyarrhythmia. Shenxian-shengmai (SXSM) is a standard Chinese formula used in bradyarrhythmia’s treat. Bradyarrhythmias, especially in right-coronary heart disorders, can occur after myocardial infarction (MI). In one study, SXSM reduced bradyarrhythmia and hearts inefficiency caused by I/R myocardial damage. SXSM raises cardiac rate while protecting against I/R myocardial damage. A report documented that SXSM reduced myocardial interstitial dilatation and structural abnormalities in myocardial cells. SXSM also protected cardiac cells against H_2_O_2_ and I/R damage by lowering ROS inside the cell. The SXSM also increased SOD activity and GSH levels in the body via increasing GCLC expression and GSH-Px activity, resulting in antiarrhythmic and cardio-protective effects (Liu et al., 2017).

## Traditional Chinese Medicine and its Clinical Trials

One of the biggest challenges in modernizing TCM is the lack of robust clinical trials. Although double-blind, controlled, and randomized studies have rarely been used to assess TCM’s efficacy in CVD therapy, some of the most commonly prescribed TCMs, such as QSYQ, Qili qiangxin Capsule, Huangqi Injection, Do xinxuekang Capsule, and Xinmailong, have already been clinically evaluated. It is commonly used in ischemic heart failure patients integrated therapy. *Radix astragali, S. miltiorrhiza, P. notoginseng*, and *Dalbergia odorifera* are all components of QSYQ. The medicine has improved left ventricular function, boosted training capacity, and reduced hospital readmission rates in small-scale clinical trials. A study of 3,505 individuals demonstrated QSYQ to be equally effective as aspirin in myocardial infarction secondary therapy (Shang et al., 2013b). In 2004, the CFDA approved the use of Qili qiangxin Capsule, another Chinese patent drug, for treating heart failure (HF). It also has *R. astragali, S. miltiorrhiza, Semen lepidii, Alismatis rhizoma*, and other Chinese herbs. *R. astragali* and *Radix aconiti carmichael* are the two most important medicinal plants (Liu et al., 2014a). A randomized, multicenter trial with 512 patients with systolic heart failure examined the *Qili qiangxin* capsule’s efficacy. After 12 weeks of treatment, the N-terminal prohormone of brain natriuretic peptide (NT-proBNP) was found in 47.95 percent of patients taking *Qili qiangxin* pills.

Compared to placebo, the Qili qiangxin tablet increased NYHA functional rating, 6-min walking distance, left ventricular ejection fraction (LVEF) range, and overall quality of life. *Qilqiangxin pills* can be used in conjunction with other medications to treat chronic heart failure (CHF). Huangqi injection is a formula of the *R. astragali* extract. There were 62 RCTs and quasi-randomized controlled studies (quasi-RCTs). The study’s methodology was determined to be flawed, and the evidence for the efficacy and safety of Huangqi injection was lacking. The benefits of hawthorn, cannabis, and curcumin on CHF have also been studied (Durst and Lotan, 2011). The Chinese FDA has approved the usage of Di’ao Xinxuekang capsules for numerous years (CFDA). Most of *Dioscorea pantheist*’s components come from its rhizomes. Its effects on angina were compared to a compound Danshan tablet in a randomized, double-blind, multicenter (Yu et al., 2014) study. The study included 733 patients. After 20 weeks of therapy, the Xinxuekang tablet lessens the proportion of patients suffering from angina pectoris. Xinxuekang has improved patients’ quality of life based on the Seattle angina questionnaire and static blood score (Jia et al., 2012). Randomized clinical research found that the Xinxuekang capsule worked better than isosorbide dinitrate in treating angina pectoris patients. Xinmailong is a proprietary Chinese medication derived from the Periplaneta Americana plant. Xinmailong showed benefits in preventing myocardial ischemia (Liu et al., 2014b). Comprehensive 15-days research with 121 patients compared Xinmailong’s effects to standard treatment. The traditional therapy group received frequent prescriptions for digitalis preparation, beta-blockers, sodium nitroprusside, and aspirin. After 7 days of routine pharmacological treatment plus Xinmailong injections, hsCRP, NT proBNP, LVEF, and the left ventricle final volume index returned to normal. After 15 days of therapy, Xinmailong outperformed standard treatment. LVEF rose from 36.9% to 46.40% following 15 days of Xinmailong therapy (LVEF was 59.7% in the regular group). To reduce angiotensin II, Xinmailong reduced the renin-angiotensin-aldosterone pathway. TCM treats individuals with syndrome differentiation. Researchers showed that after 28 days of treatment with TCM, patients’ LVEF improved more than the placebo group. Patients could choose from a variety of Chinese herbal mixtures to treat their problems. When compared to the placebo group, the TCM symptom evaluations improved dramatically. Only one TCM-treated group patient developed atrial fibrillation (AF) (Luo et al., 2015). The study found that Chinese herbal therapy enhanced heart function in those with CHF and was safe. Two more trials using Nuanxin capsules and Shencao tongmai granules found that the TCM syndrome rates of patients with CHF were lower in the Chinese herbal medicine group than in the placebo group. The symptoms and indications of CHF patients were considerably reduced when treated with Chinese herbal medicine (Wang et al., 2012). The quantities of bioactive chemicals can fluctuate between capsules, complicating clinical trials (Li et al., 2013). Given the inconsistency of substances in formulations or tablets, clinical research should be viewed with caution and cautious hope. Clinical research can study how TCM multi-compounds affect heart failure syndrome (Tang and Huang, 2013).

## Adverse Events and Toxic Reactions of TCM

Ginseng extracts have been shown to have minor adverse effects in several *in vitro* and *in vivo* experiments, as well as human clinical studies. Following lengthy durations of administration of large dosages of Ginseng extracts, just a few adverse effects were documented. Morning diarrhea, skin eruptions, anxiety, insomnia, hypertension, edema, reduced appetite, depression, and hypotension were among the symptoms (Shaito et al., 2020). Ginseng supplements have also been linked to many clinically significant adverse cardiovascular events. There have been several instances of extended Ginseng usage or misuse leading to possible cardiovascular adverse effects such as elevated BP, long QT syndrome, or AF (Paik and Lee, 2015). Lungs patients who received 4 weeks 1.5 g/day curcumin treatment showed some adverse events, including gastrointestinal problems (stomachache, constipation), and headache without any major toxic effect and adverse reaction (Soleimani et al., 2018). Clinical trials and experimental data showed that *Dioscorea* species could cause toxicity, specifically in the liver (Zhao et al., 2018b). Conventionally, the safe use of TCM practice relies on correct pharmacognostic identification, good agricultural and manufacturing practices based on pharmacopoeia standards with TCM-guided clinical prescribing. To avoid incidents of adverse reactions to TCM, a good practice environment, and quality assurance are the key factors for global acceptance of their evidence-based usefulness in healthcare (Chan et al., 2015; Zhao et al., 2020).

### Challenges and Flaws Holding TCM Back

Although TCM has had worldwide applications (Franz, 2011), Western scientist often criticizes or even reject this primeval art of TCM because it consists of numerous ingredients with myriad chemical compounds. If not impossible, it becomes difficult to demonstrate the therapeutic potential of TCM against CVD. Current TCM treatments available in China do not go through the same strict approval processes as Western drugs, thus their safety and efficacy are not guaranteed (Tang et al., 2018). In China most TCM clinical trials were done by TCM physicians, while Western countries have relatively few TCM medicines and experts, their studies are of poor quality, and the findings drawn are not accepted by Western culture (Bartecchi, 2006; Ekor, 2014; Chen et al., 2019). The current randomized controlled studies on TCM for CVD are flawed and methodological weaknesses are still there, with small sample numbers and mixed results, making it impossible to make firm conclusions regarding TCM’s true benefits and risks (Hao et al., 2017; Zhang et al., 2019). Most of the TCM studies, especially early studies, are compromised by methodological flaws. For example, more than 2000 clinical trials can be found for Xiao Yao San (a classic TCM formula used for over 2000 years), and only two have met the standard of double/single-blinded randomized controlled trials (Zhou et al., 2019). To clarify these doubts about TCM and improve its current state, high-quality RCTs of TCM therapy are needed (Hao et al., 2017). For the continuous development of TCM in CVD therapy set clinical endpoints, the safety control, strong efficacy evaluation and development of TCM in a modernized scientific manner is needed to minimize the safety risks and provide scientific proof and clinical evaluation (Zhou et al., 2019; Hu et al., 2021).

## Conclusion and Future Perspective

Therapy of the TCM has particular advantages in the cure of CVD due to its antioxidant components. TCM has shown significant progress in very recent years. TCM effectively amend the symptoms associated with CVD and therefore improve the patient’s quality. TCM has minimal side effects but with numerous and significant therapeutic effects and is not dependent on the drug. CVD therapy and the use of TCM against this disease has a comprehensive vision, and it is quite worthy for further promotion and development. Furthermore, there is a clear and rigorously need for well‐designed large‐scale randomized‐controlled trials (RCTs) together with mechanistic studies to allow proper evaluation of effect, potential therapeutic benefit, and major adverse cardiovascular events of TCM in patients with CVD.
